# Prevalence and Clinical Associations of Systemic Sclerosis-Related Autoantibodies: A Nationwide Reuma.pt Cohort Study

**DOI:** 10.3390/antib15050085

**Published:** 2026-09-15

**Authors:** Carolina Mazeda, Eduardo Dourado, Raquel Freitas, Patrícia Martins, Liliana Saraiva, Tânia Santiago, Francisca Guimarães, Emanuel Costa, Diogo Esperança Almeida, Sara Dinis, Ana Sofia Pinto, Alexandra Daniel, Inês Genrinho, Maura Couto, Marília Rodrigues, Maria João Salvador, Ana Catarina Duarte, Ana Cordeiro, Maria José Santos, João Eurico Fonseca, Catarina Resende, Inês Cordeiro

**Affiliations:** 1 Department of Rheumatology, Unidade Local de Saúde da Região de Aveiro, 3810-164 Aveiro, Portugal; 2 Centro de Investigação em Reumatologia de Aveiro, Centro Académico Clínico Egas Moniz, 3810-164 Aveiro, Portugal; 3 EpiDoc Unit, NOVA Medical School, NOVA University Lisbon, 1169-056 Lisboa, Portugal; 4 Unidade de Investigação em Reumatologia, Instituto de Medicina Molecular, Faculdade de Medicina, Universidade de Lisboa, CAML, 1649-028 Lisboa, Portugal; 5 Department of Rheumatology, Unidade Local de Saúde de Almada Seixal, 2805-267 Almada, Portugal; 6 Department of Rheumatology, Unidade Local de Saúde Viseu Dão-Lafões, 3504-509 Viseu, Portugal; 7 Department of Rheumatology, Unidade Local de Saúde de Coimbra, 3000-075 Coimbra, Portugal; 8 Institute for Clinical and Biomedical Research, Faculdade de Medicina, Universidade de Coimbra, 3000-548 Coimbra, Portugal; 9 Department of Rheumatology, Unidade Local de Saúde do Alto Minho, 4990-078 Ponte de Lima, Portugal; 10 Department of Rheumatology, Unidade Local de Saúde de Braga, 4710-243 Braga, Portugal; 11 Department of Rheumatology, Unidade Local de Saúde da Guarda, 6300-858 Guarda, Portugal; 12 Department of Rheumatology, Unidade Local de Saúde da Região de Leiria, 2410-197 Leiria, Portugal; 13 Pain Unit, Unidade Local de Saúde da Região de Aveiro, 3810-164 Aveiro, Portugal; 14 Department of Rheumatology, Unidade Local de Saúde de Lisboa Norte, Centro Académico de Medicina de Lisboa (CAML), 1649-035 Lisboa, Portugal

**Keywords:** systemic sclerosis, systemic sclerosis-specific antibodies, multicentre register

## Abstract

**Background:** Autoantibodies are central to the diagnosis and risk stratification of systemic sclerosis (SSc), but their prevalence and clinical associations may vary across populations. This study aimed to evaluate the prevalence and immuno-clinical associations of different autoantibodies in the Rheumatic Diseases Portuguese Register systemic sclerosis cohort. **Methods:** This was a multicentre observational registry-based study that used data from the Reuma.pt SSc module. Patients were divided according to autoantibody status, and associations between autoantibody expression and clinical data were assessed using appropriate statistical tests, with Bonferroni correction applied for multiple comparisons. Multivariable binary logistic regression was performed for clinically relevant outcomes, with adjustment for sex, age at diagnosis and disease duration. **Results:** A total of 1080 patients were included, 87.5% female, with a mean age at last evaluation of 60.2 ± 14.6 years and a mean disease duration of 12.4 ± 10.0 years. Limited cutaneous SSc was the most frequent clinical category (57.4%), followed by diffuse cutaneous SSc (17.7%), very early diagnosis of systemic sclerosis (VEDOSS; 12.3%), overlap syndromes (9.8%) and SSc sine scleroderma (2.8%). Antinuclear antibodies were present in 93.4% of patients. Anti-centromere antibodies (ACAs) were the most frequent SSc-specific autoantibodies (54.6%), followed by topoisomerase I antibodies (ATAs; 21.8%). ACA positivity was associated with limited cutaneous disease; older age at diagnosis; and lower frequency of interstitial lung disease (ILD), myositis and flexion contractures. ATA positivity was associated with diffuse cutaneous disease, male sex, higher modified Rodnan skin score (mRSS), digital ulcers, flexion contractures, oesophageal involvement and ILD. Among less frequent autoantibodies, anti-Pm/Scl antibodies were associated with myositis, joint involvement, calcinosis and ILD; anti-U1RNP antibodies with younger age, MCTD overlap, myositis and joint involvement; anti-RNA polymerase III antibodies (ARAs) with scleroderma renal crisis and higher mRSS; anti-U3RNP antibodies with diffuse cutaneous disease and renal involvement; and anti-Ku antibodies with overlap syndromes. **Conclusions:** In this large real-world SSc cohort, autoantibody status was strongly associated with distinct clinical phenotypes, confirming its value for disease stratification. While most established immuno-clinical associations were reproduced, some differences from international cohorts were observed, supporting the need for population-specific validation of autoantibody associations in SSc.

## 1. Introduction

Systemic sclerosis (SSc) is a heterogeneous systemic autoimmune rheumatic disease (SARD) characterised by immune dysregulation, vasculopathy and progressive fibrosis of the skin and internal organs [1,2]. One of the typical features of SSc is the occurrence of disease-specific antibodies, which are detected in the vast majority of patients and represent key markers for diagnosis and prognostic assessment [1,3]. The most commonly observed are anti-centromere antibodies (ACAs), anti-topoisomerase I antibodies (ATAs), and anti-RNA polymerase III antibodies (ARAs), which have been consistently linked to different clinical phenotypes [4]. ACAs are typically associated with a more indolent disease course, limited cutaneous involvement (lcSSc), and vascular complications [3,4,5,6], whereas ATAs are associated with interstitial lung disease (ILD) and more severe fibrotic involvement [7,8]. ARAs are associated with rapidly progressive skin disease, scleroderma renal crisis (SRC), and an increased risk of malignancy [9]. In addition to these classical antibodies, a broader spectrum of less common autoantibodies, including anti-Pm/Scl, anti-U1RNP, anti-U3RNP, anti-Th/To, anti-Ku, and anti-NOR90, has emerged as clinically relevant. These autoantibodies are increasingly recognised as defining distinct immuno-clinical clusters frequently associated with overlap syndromes or specific patterns of organ involvement, such as myositis, pulmonary hypertension, and gastrointestinal disease [3,10,11,12,13,14]. However, the clinical relevance of many of these associations remains incompletely understood, and findings are often inconsistent across studies.

Large population-based and registry studies have reinforced the central role of immunological profiling in SSc. Data from international registries, particularly the European Scleroderma Trials and Research (EUSTAR) cohort, have demonstrated that autoantibody status is a major determinant of organ involvement, disease trajectory, and survival [2,15,16]. At the same time, studies across different populations have demonstrated variability in the prevalence of autoantibody subsets and their clinical associations, particularly for less prevalent antibodies [17]. Real-world cohorts provide a unique opportunity to explore these questions in a comprehensive manner. The Rheumatic Diseases Portuguese Register (Reuma.pt), a nationwide multicentre registry, provides a unique opportunity to evaluate immuno-clinical associations in a large, well-characterised real-world cohort of patients with SSc [18,19]. Analysis of this cohort may help to clarify the prevalence and clinical relevance of different autoantibody profiles and to determine whether the traditional concept of serological subsets in SSc is valid in this population, while also identifying population-specific features with potential implications for clinical practice.

The aim of this study was to evaluate the prevalence and immuno-clinical associations of different autoantibodies in the Reuma.pt SSc cohort.

## 2. Methods

### 2.1. Study Design and Population

This was a multicentre observational registry-based study using data from the Reuma.pt SSc module. We included adult patients registered in the SSc module of Reuma.pt between August 2015 and February 2021 who were diagnosed as having SSc by their treating rheumatologist. Exclusion criteria included patients who had not consented for their data to be used for scientific investigation, age of onset before 18 years old, and key missing data (e.g., disease subtype).

### 2.2. Data Collection and Management

Data were prospectively collected into the Reuma.pt/SSc module by the assisting rheumatologists and were subsequently exported into an anonymised Microsoft Excel file to be analysed.

We extracted demographic data (sex, age, age at diagnosis, and ancestry), date of the first non-Raynaud symptom, date of SSc diagnosis, disease classification within the SSc spectrum [lcSSc, dcSSc, very early diagnosis of systemic sclerosis (VEDOSS), SSc sine scleroderma (ssSSc), and overlap syndrome], fulfilment of the 2013 European Alliance of Associations for Rheumatology (EULAR)/American College of Rheumatology (ACR) classification criteria [20], cumulative clinical manifestations [Raynaud’s phenomenon (RP), skin thickening, telangiectasias, digital ulcers (DUs), fingertip pitting scars, calcinosis, tendon friction rubs, joint involvement, myositis, gastrointestinal, heart, pulmonary or SRC], modified Rodnan skin score (mRSS), immunological features [antinuclear antibody (ANA), ATA, ACA, ARA, anti-Th/To, anti-U3 RNP, anti-Pm/Scl, anti-Ku, anti-U1 RNP, anti-U11/U12 RNP antibody positivity], and type of nailfold capillaroscopy pattern (most recent report). Clinical manifestations and organ involvement were recorded according to the treating rheumatologist’s clinical judgement, based on available clinical, laboratory, functional and imaging data, as applicable.

### 2.3. Study Groups

Patients were divided according to the presence or absence of each autoantibody. For each autoantibody, positive patients were compared with all patients who tested negative for that same autoantibody and had available data for the variable under analysis.

### 2.4. Statistical Analysis

Descriptive statistics were used to summarise cumulative clinical and immunological features, comorbidity and treatment characteristics. Continuous variables were presented as means and standard deviations (SDs) or medians and interquartile ranges (IQRs) and categorical variables as absolute and relative frequencies.

Univariate analyses were performed to assess associations between autoantibody expression and sociodemographic and clinical variables. Comparisons between groups were conducted using the chi-square test or Fisher’s exact test for categorical variables and the Mann–Whitney U test for continuous variables, as appropriate. Effect sizes for categorical variables were expressed as odds ratios (ORs) with 95% confidence intervals (95% CIs). To account for multiple comparisons, a Bonferroni correction was applied. Accordingly, associations were considered statistically significant at *p* ≤ 0.002 and were defined as definite associations, while those with *p* ≤ 0.05 were considered likely associations. Due to the small number of positive patients in some autoantibody groups, associations involving rare autoantibodies were interpreted as exploratory.

As an additional adjusted analysis, multivariable binary logistic regression was performed. Autoantibody profile was categorised into three groups: ACA, ATA and a non-ACA/ATA group comprising patients positive for other SSc-related autoantibodies, as well as patients without any detectable autoantibody. Patients positive for more than one autoantibody-defining group were excluded from this analysis. Clinical manifestations to be evaluated as the dependent variable of multivariable analyses were defined a priori as variables that, in univariate analysis, yielded associations either not previously described in the literature or opposite to established immuno-clinical patterns. Each selected clinical manifestation was analysed as a binary dependent variable. In each model, autoantibody group was entered as the variable of interest, and models were adjusted for sex, age at diagnosis and disease duration. Results are reported as adjusted ORs with 95% CIs. For outcomes with few events, Firth penalised logistic regression was additionally applied. Collinearity was assessed using the variance inflation factor (VIF). All analyses were performed in the full cohort and repeated in the subgroup of patients fulfilling the 2013 ACR/EULAR classification criteria for sensitivity analysis. Standard statistical analyses were performed using IBM SPSS Statistics, version 27; Firth penalised logistic regression was performed using Python version 3.12. 

### 2.5. Ethical Considerations

This study was conducted according to the principles of the Declaration of Helsinki (revised in Helsinki—2024) and was approved by the Ethics Committee of Centro Académico de Medicina de Lisboa (approval number: 416/19), and the Coordinating and Scientific Board of Reuma.pt. Reuma.pt was approved by the National Board for Personal Data Protection and local ethics committees. All included patients signed an informed consent form for registration and use of their pseudoanonymised data.

## 3. Results

### 3.1. Cohort Demographics

A total of 1080 patients were included. Most patients were female (N = 945/1080, 87.5%), with a predominance of white European ancestry (N = 599/643, 93.2%), while African ancestry accounted for 4.7% of patients. The mean age at last evaluation was 60.2 ± 14.6 years, with a mean disease duration of 12.4 ± 10.0 years and a mean diagnostic delay of 5.0 ± 8.2 years (Table 1).

### 3.2. Cohort Clinical Characterisation

Regarding disease classification within the SSc spectrum, lcSSc was the most frequent clinical category (N = 620/1080, 57.4%), followed by dcSSc (N = 191/1080, 17.7%), very early diagnosis of SSc (VEDOSS) (N = 133/1080, 12.3%), overlap syndromes (N = 106/1080, 9.8%) and ssSSc (N = 30/1080, 2.8%).

Skin involvement with skin thickening was present in 75.1% (N = 789/1050) of patients, with a mean modified Rodnan skin score (mRSS) at the last appointment of 4.7 ± 7.1. Musculoskeletal manifestations included joint involvement (arthralgia or arthritis) in 39.9% (N = 400/1002) and myositis in 6.3% (N = 62/984) of patients. Vascular involvement was common, with Raynaud’s phenomenon (RP) in 93.4% (N = 978/1047), digital ulcers (DUs) in 35.1% (N = 365/1040), pitting scars in 31.0% (N = 287/927), and telangiectasia in 48.9% (N = 493/1008). ILD was present in 33.2% (N = 289/870) of patients, while pulmonary arterial hypertension (PAH), confirmed by right heart catheterisation, was observed in 1.9% (N = 19/1021). Gastrointestinal involvement was frequent, particularly oesophageal involvement (N = 354/971, 36.5%), while cardiac involvement and SRC were observed in 7.6% (N = 73/966) and 1.9% (N = 18/961) of patients, respectively.

### 3.3. Autoantibody Prevalence

Antinuclear antibodies were detected in 93.4% (N = 963/1031) of patients. Among SSc-specific autoantibodies, ACAs were the most prevalent (N = 569/1043, 54.6%), followed by ATAs in 21.8% (N = 226/1036). Less frequent autoantibodies included anti-Pm/Scl (N = 31/665, 4.7%), anti-U1-RNP (N = 28/693, 4.0%), ARA (N = 24/806, 3.0%), anti-U3-RNP (N = 16/643, 2.5%), anti-Th/To (N = 13/606, 2.1%), anti-Ku (N = 12/633, 1.9%), and anti-U11/U12-RNP (N = 3/613, 0.5%). Overall, 26 patients were positive for more than one autoantibody-defining group.

### 3.4. Autoantibody Associations

**Figure 1:**
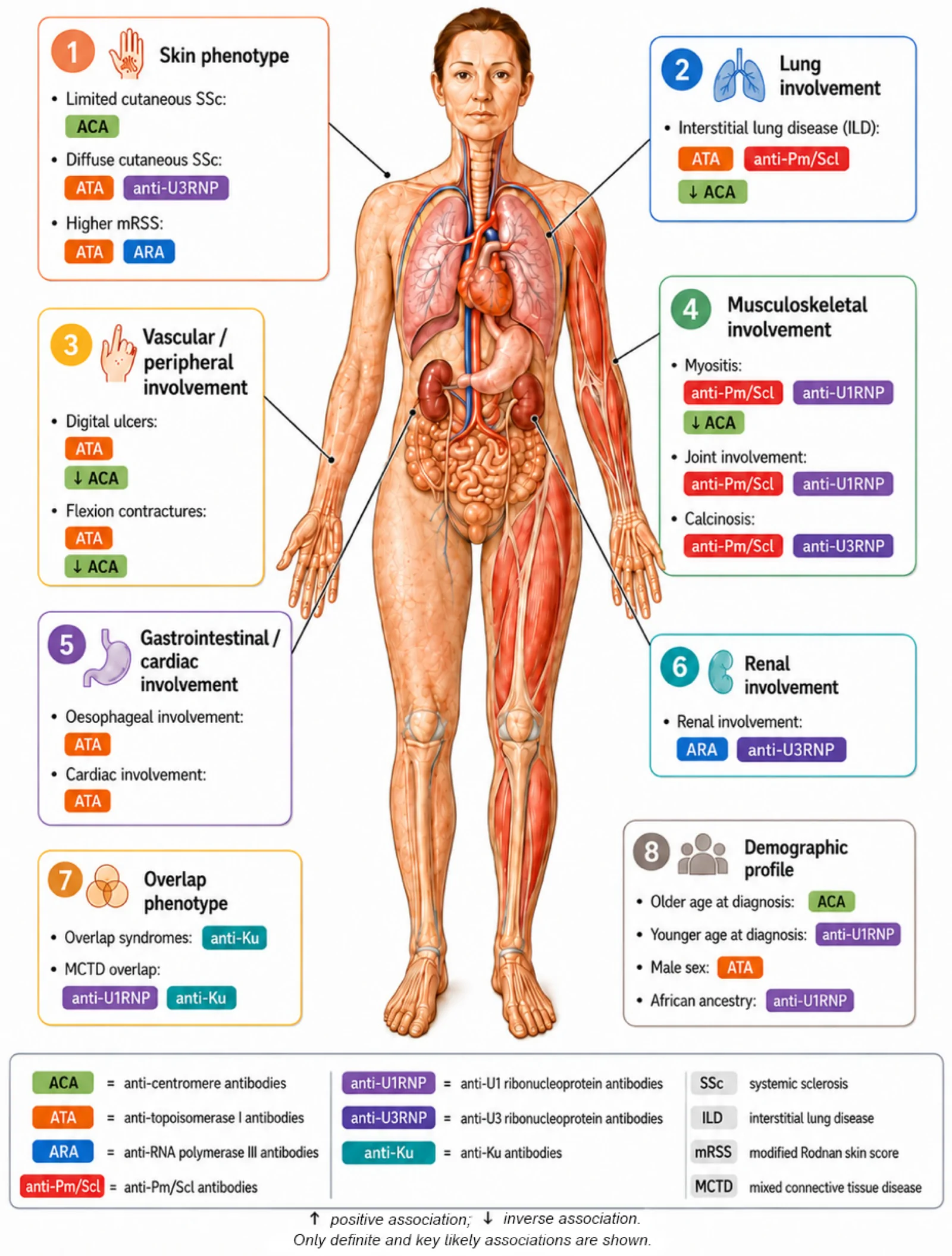
Key immuno-clinical associations from the Rheumatic Diseases Portuguese Register systemic sclerosis cohort.

The associations between autoantibody positivity and demographic and clinical characteristics are summarised in Table 2 and visually represented in Figure 1. A comprehensive overview of all tested associations is provided in the online Supplementary Table S1.

#### 3.4.1. Anticentromere Autoantibodies

ACA positivity was strongly associated with lcSSc (OR: 4.17, 95% CI: 3.21–5.42, *p* < 0.001). ACA-positive patients were older at symptom onset and diagnosis (median: 50.4 vs. 45.9 and 54.6 vs. 49.1 years, respectively, *p* < 0.001) and had a slightly longer diagnostic delay (median: 2.0 vs. 1.5 years, *p* = 0.031). These patients were significantly more commonly females (OR: 4.52, 95% CI: 2.96–6.92, *p* < 0.001), of white European ancestry (OR: 2.70, 95% CI: 1.38–5.31, *p* = 0.003), with VEDOSS (OR: 1.62, 95% CI: 1.11–2.36, *p* = 0.012) or isolated sclerodactyly (OR: 1.86, 95% CI: 1.41–2.46, *p* < 0.001). Conversely, ACA positivity was inversely associated with SSc in overlap with other connective tissue diseases (OR: 0.26, 95% CI: 0.16–0.42, *p* < 0.001), particularly with polymyositis (OR: 0.08, 95% CI: 0.02–0.34, *p* < 0.001), dermatomyositis (OR: 0.08, 95% CI: 0.01–0.64, *p* = 0.004) and mixed connective tissue disease (MCTD) overlap (OR: 0.06, 95% CI: 0.01–0.26, *p* < 0.001). Musculoskeletal and vascular involvements were less frequent, including flexion contractures (OR: 0.19, 95% CI: 0.10–0.33, *p* < 0.001), myositis (OR: 0.13, 95% CI: 0.07–0.27, *p* < 0.001), digital ulcers (OR: 0.65, 95% CI: 0.50–0.84, *p* = 0.001), and pitting scars (OR: 0.69, 95% CI: 0.52–0.91, *p* = 0.008). ACAs were also associated with lower rates of oesophageal involvement (OR: 0.75, 95% CI: 0.58–0.98, *p* = 0.037) and pulmonary involvement with ILD (OR: 0.21, 95% CI: 0.16–0.29, *p* < 0.001).

#### 3.4.2. Anti-Topoisomerase Autoantibodies

ATA positivity was strongly associated with dcSSc (OR: 11.26, 95% CI: 7.83–16.20, *p* < 0.001) and higher mRSS score in the last evaluation (median: 6 vs. 1, *p* < 0.001). Patients were younger at diagnosis (median: 49.9 vs. 53.3, *p* = 0.036) and were more frequently male (OR: 2.00, 95% CI: 1.34–2.99, *p* = 0.001). ATA-positive patients had increased frequencies of flexion contractures (OR: 4.17, 95% CI: 2.55–6.80, *p* < 0.001), tendon friction rubs (OR: 3.48, 95% CI: 1.51–8.02, *p* = 0.004), digital ulcers (OR: 2.00, 95% CI: 1.47–2.71, *p* < 0.001) and pitting scars (OR: 2.00, 95% CI: 1.45–2.76, *p* < 0.001). ILD (OR: 6.15, 95% CI: 4.34–8.72, *p* < 0.001), oesophageal involvement (OR: 1.70, 95%CI, 1.25–2.33, *p* = 0.001), and cardiac involvement (OR: 1.89, 95% CI: 1.12–3.20, *p* = 0.016) were more prevalent. Additionally, ATA was associated with an active capillaroscopic pattern (OR: 1.68, 95% CI: 1.05–2.68, *p* = 0.029).

#### 3.4.3. Less Prevalent SSc-Specific and Associated Autoantibodies

ARA positivity was associated with SRC (OR: 9.75, 95% CI: 2.53–37.57, *p* = 0.007) and higher mRSS (median: 5 vs. 2, *p* = 0.014).

In patients with anti-Th/To positivity, male sex (OR: 4.13, 95% CI: 1.32–12.94, *p* = 0.023), polymyositis overlap (OR: 10.58, 95% CI: 2.07–54.07, *p* = 0.025), and myositis (OR: 5.31, 95% CI: 1.39–20.31, *p* = 0.033) were more common.

Anti-U1RNP-positive patients were significantly younger at diagnosis (median: 36.7 vs. 53.9, *p* < 0.001) and more frequently of African ancestry (OR: 6.88, 95% CI: 2.04–23.25, *p* = 0.007). Anti-U1RNP was strongly associated with MCTD overlap (OR: 147.83, *p* < 0.001), myositis (OR: 7.87, 95% CI: 3.20–19.34, *p* < 0.001), and joint involvement (OR: 2.65, 95% CI: 1.19–5.87, *p* = 0.013).

Anti-Pm/Scl positivity was associated with male sex (OR: 2.79, 95% CI: 1.24–6.28, *p* = 0.010), calcinosis (OR: 2.94, 95% CI: 1.21–7.14, *p* = 0.023), joint involvement (OR: 2.45, 95% CI: 1.14–5.27, *p* = 0.019), myositis (OR: 5.96, 95% CI: 2.37–15.00, *p* = 0.001), and ILD (OR: 2.20, 95%CI, 1.03–4.66, *p* = 0.037).

Patients positive for anti-U3RNP more commonly had dcSSc (OR: 6.32, 95% CI: 2.30–17.34, *p* = 0.001), calcinosis (OR: 3.55, 95% CI: 1.08–11.68, *p* = 0.050), and scleroderma renal crisis (SRC) (OR: 13.55, 95% CI: 2.51–73.12, *p* = 0.018).

Finally, anti-Ku antibodies were associated with overlap syndromes, particularly systemic lupus erythematosus (SLE) (OR: 15.33, 95% CI: 2.88–81.47, *p* = 0.014) and MCTD (OR: 7.11, 95% CI: 1.45–34.95, *p* = 0.047), without clear associations with SSc-specific organ involvement.

#### 3.4.4. Patients Without Detectable SSc-Related Autoantibodies

Overall, 193 patients had no detectable SSc-related autoantibodies. Among these, 123 were ANA-positive only, 38 were ANA-negative and 32 had missing or unknown ANA status. This subgroup was clinically heterogeneous, 45.6% were classified as lcSSc, 22.3% as dcSSc, 11.9% as VEDOSS, 18.7% as overlap syndromes and 1.6% as ssSSc. Regarding organ involvement, ILD was present in 38.7%, oesophageal involvement in 42.7%, PAH in 2.3%, calcinosis in 13.0%, myositis in 14.7% and SRC in 0.7%. Compared with patients positive for at least one SSc-related autoantibody, patients without detectable SSc-related autoantibodies more frequently had overlap syndromes (18.7% vs. 7.9%; *p* < 0.001) and myositis (14.7% vs. 4.6%; *p* < 0.001). The proportions of patients classified as VEDOSS (11.9% vs. 12.4%; *p* = 0.853) and fulfilling the 2013 ACR/EULAR classification criteria (77.5% vs. 78.1%; *p* = 0.876) were similar between groups. No clear differences were observed regarding ILD, PAH, oesophageal involvement, calcinosis or SRC.

#### 3.4.5. Multivariable Analyses

A total of 1054 patients were included in the multivariable analyses (ACA, n = 553; ATA, n = 211; non-ACA/ATA group, n = 290, including 193 patients without detectable SSc-related autoantibodies); 26 patients positive for more than one group were excluded. The clinical manifestations selected for the adjusted analysis were oesophageal involvement, calcinosis, PAH and SRC. No relevant collinearity was identified in any model (all VIFs < 1.2), and Firth estimates for the rare outcomes were concordant with the conventional models.

Compared with ACA, ATA positivity was independently associated with oesophageal involvement (adjusted OR: 1.92; 95% CI: 1.30–2.83; *p* = 0.001), whereas non-ACA/ATA group did not differ significantly from ACA for this outcome (adjusted OR: 1.13; 95% CI: 0.76–1.67). No significant associations were found between the groups regarding SRC, calcinosis or PAH (Table 3).

In the subgroup fulfilling the 2013 ACR/EULAR classification criteria (n = 786; ACA, n = 405; ATA, n = 188; non-ACA/ATA group, n = 193), the association between the ATA group and oesophageal involvement was also significant (adjusted OR: 1.58; 95% CI: 1.03–2.41; *p* = 0.036), with no significant associations for the remaining outcomes.

## 4. Discussion

In this large nationwide cohort of patients with SSc from the Reuma.pt registry, autoantibody expression was strongly associated with distinct clinical phenotypes, supporting its central role in disease stratification. The prevalence and clinical associations of several autoantibodies in our cohort were broadly consistent with previous reports, although relevant population-specific differences were identified [2,5,15].

ACA was the most prevalent autoantibody in the cohort (54.6%), exhibiting a higher prevalence than that typically reported in other SSc populations (25.3–30.3%) [5,22,23]. This may be partly explained by the high proportion of patients of white European ancestry, as ACA positivity has been described more frequently in white and female patients [5,6,7,24,25]. In line with previous reports, ACA positivity was associated with lcSSc, older age at diagnosis, lower mRSS, and a lower frequency of ILD and digital ulcers [2,5,8,26,27]. However, contrary to some prior studies, ACA positivity was associated with lower rates of oesophageal involvement and was not associated with calcinosis or PAH in our cohort [7,22]. Given that our cohort included a substantial proportion of VEDOSS, the high prevalence of ACA positivity is plausible, as VEDOSS cohorts are enriched for SSc-specific antibodies, particularly ACAs and ATAs [28]. In addition, oesophageal dysfunction has been shown to occur already during the VEDOSS stage [29], which may reduce the ability to detect ACA-specific associations in mixed cohorts enriched for early disease presentations. Although calcinosis has been associated with ACAs in some cohorts, this relationship has not been consistently reproduced across populations and study designs, and differences in disease duration, cohort composition and methods of clinical assessment may partly explain the absence of association observed in our real-world cohort [30,31]. The overall prevalence of PAH in our cohort was low. Importantly, PAH cases were only considered when confirmed by right heart catheterisation, which increases diagnostic specificity but may underestimate the true prevalence.

The prevalence of ATA positivity (21.8%) was within the range reported in the literature (20.5–36.0%) [5,22,32]. As expected, ATA positivity was associated with dcSSc, male sex, younger age at diagnosis, and a more severe phenotype, including digital ulcers, flexion contractures, tendon friction rubs, ILD and cardiac involvement [2,7,8,26,33]. These findings reinforce the role of ATA as a marker of fibrotic and progressive disease. Importantly, the association between ATA and oesophageal involvement was maintained after adjustment for sex, age at diagnosis and disease duration, and remained significant in the subgroup fulfilling the 2013 ACR/EULAR classification criteria. Previous studies have reported inconsistent associations between ATA and oesophageal involvement, likely reflecting differences in cohort composition, disease stage and the definition of gastrointestinal involvement [34,35,36]. In the recent SPRING-SIR registry, ATAs were not enriched among patients with more extensive GI symptom involvement, as ATAs did not emerge as an independent predictor of symptom extent [37]. Notably, ATA positivity was also associated with an active scleroderma pattern on capillaroscopy and pitting scars, supporting a potential link between ATA positivity, active microvascular damage and peripheral vasculopathy. Unlike some previous reports [38], ATA-positive patients did not show increased renal involvement in this cohort. This discrepancy is plausible given the marked heterogeneity in the reported frequency of renal manifestations across cohorts, particularly SRC, with pooled prevalence estimates around 4%, and the fact that ATA has not consistently emerged as an independent predictor of renal crisis in large registry-based analyses [39,40].

ARA positivity was uncommon, with a lower prevalence (3.0%) than that reported in the recent meta-analysis (9%) by Elhannani et al. [9]. Nevertheless, ARA positivity was associated with SRC and higher mRSS, consistent with its known association with scleroderma renal crisis and aggressive skin disease [9]. The low number of ARA-positive patients may have limited the detection of other associations, including malignancy, gastric involvement (such as gastric antral vascular ectasia) and cardiac involvement.

Anti-Pm/Scl prevalence in our cohort (4.7%) was similar to that reported in several previous cohorts [14,22]. Anti-Pm/Scl positivity was associated with myositis, joint involvement, and calcinosis, consistent with an overlap phenotype. It was also associated with ILD, in line with large registry analyses showing increased ILD frequency in anti-Pm/Scl-positive SSc and supporting the clinical relevance of pulmonary assessment in this overlap phenotype [14].

Anti-U1RNP was associated with younger age, African ancestry, MCTD overlap, joint involvement and myositis, in agreement with recent data suggesting a distinct overlap phenotype in SSc [11,41,42]. Anti-U3RNP was associated with dcSSc, calcinosis and SRC in our cohort, but we did not observe an association with ILD or PAH, differing from previous reports linking this antibody to severe cardiopulmonary disease [10,20]. Anti-Ku was mainly associated with overlap syndromes in our study, consistent with international cohort data [13,43]. Overall, the absence of significant cardiopulmonary associations for anti-U3RNP, anti-Th/To and anti-U1RNP in our cohort should be interpreted with caution. These findings are likely influenced by the low number of patients with these autoantibodies, the low prevalence of PAH and the predominance of white European ancestry, particularly relevant for anti-U3RNP [43]. In addition, the cross-sectional design may have limited detection of late-onset cardiopulmonary complications, especially in anti-Th/To patients, in whom these manifestations may develop later during the disease course. Differences in referral patterns and diagnostic practices may also have contributed. Furthermore, anti-Th/To and other rare autoantibodies have only become more widely available in routine clinical practice in recent years; consequently, patients included earlier in the registry may not have been systematically tested, potentially leading to underestimation of their prevalence and limiting the ability to detect robust clinical associations.

The inclusion of SSc overlap syndromes may have contributed to some of the observed associations, particularly for anti-Pm/Scl, anti-U1RNP and anti-Ku antibodies, which are frequently enriched in overlap phenotypes. Nevertheless, these patients represent a clinically relevant subset of the SSc spectrum, and their inclusion reflects the real-world design of the Reuma.pt SSc registry.

Our findings confirm that most established immuno-clinical associations apply to the Portuguese SSc population but also demonstrate that these relationships may not be universal. Differences between our cohort and previous studies may reflect population-specific factors, particularly ancestry, as well as differences in study design, disease duration, referral patterns, and registry-based data collection [4,17].

This study provides robust real-world data from a large nationwide multicentre cohort. However, some limitations should be acknowledged. First, the number of patients with specific manifestations, particularly PAH and SRC, was low, as was the number of patients with rarer autoantibodies. The absence of adjusted associations with PAH and SRC should be interpreted cautiously, given the low number of events and limited statistical power for these outcomes, although Firth penalised regression yielded concordant results. Second, the cross-sectional design precludes assessment of temporal relationships and disease progression, and the under-reporting of some clinical manifestations cannot be excluded in a registry-based study. Variability in autoantibody testing across centres may have influenced the prevalence of less common antibodies. Furthermore, these rare autoantibodies have only become more widely available in routine clinical practice in recent years; consequently, patients included earlier in the registry may not have been systematically tested, potentially leading to underestimation of their prevalence and limiting the ability to detect robust clinical associations.

In conclusion, this large Portuguese SSc cohort confirms the central role of autoantibodies in defining disease phenotype, while highlighting relevant population-specific differences, particularly regarding ACA, ATA, and anti-Pm/Scl-associated manifestations. From a practical perspective, ATA and anti-Pm/Scl profiles may support closer pulmonary surveillance, whereas the associations observed between ARA and anti-U3RNP positivity and SRC suggest that these profiles may help identify patients in whom closer attention to renal manifestations is warranted. However, these results should be interpreted cautiously, given the low absolute number of patients with renal involvement and with these rarer autoantibodies. Overall, these findings reinforce the importance of autoantibody profiling as one of the most clinically informative tools for disease stratification in SSc, while also supporting the need for population-specific validation, standardised autoantibody testing, and longitudinal analyses to better define the prognostic implications of rarer autoantibody subsets.

## Figures and Tables

**Table 1:** Demographic and clinical data from the 1080 patients included in the study.

Variable	NPAD	Total
Demographic data		
Females, N (%)	1080	945 (87.5)
Ancestry		
White European, N (%)	643	599 (93.2)
African, N (%)	643	30 (4.7)
Non-European white, N (%)	643	7 (1.1)
Mixed African and European, N (%)	643	5 (0.8)
Romani, N (%)	643	1 (0.2)
Asian, N (%)	643	1 (0.2)
Age at the symptom onset, mean (SD)	824	47.3 (15.5)
Age at diagnosis, mean (SD)	927	52.3 (14.7)
Age at the last appointment, mean (SD)	1080	60.2 (14.6)
Diagnosis delay, mean (SD)	815	5.0 (8.2)
Disease duration, mean (SD)	824	12.4 (10.0)
Death, N (%)	1080	79 (7.3)
Disease clinical categories		
Limited cutaneous SSc, N (%)	1080	620 (57.4)
Diffuse cutaneous SSc, N (%)	1080	191 (17.7)
SSc sine scleroderma, N (%)	1080	30 (2.8)
Very early diagnosis of SSc, N (%)	1080	133 (12.3)
Overlap syndromes, N (%)	1080	106 (9.8)
Overlap with polymyositis, N (%)	1079	22 (2.0)
Overlap with dermatomyositis, N (%)	1079	12 (1.1)
Overlap with systemic lupus erythematosus, N (%)	1080	22 (2.0)
Overlap with Sjögren’s syndrome, N (%)	1077	24 (2.2)
Overlap with rheumatoid arthritis, N (%)	1080	9 (0.8)
Mixed connective tissue disease, N (%)	1080	29 (2.7)
Classification criteria		
2013 ACR/EULAR classification criteria, N (%)	1035	807 (78.0)
1980 ACR classification criteria, N (%)	904	548 (60.6)
Clinical manifestations		
Skin disease		
Skin thickening, N (%)	1050	789 (75.1)
Skin thickening proximal to the MCP in any location, N (%)	909	432 (47.5)
Skin thickening proximal to the MCP in hand, N (%)	1008	411 (40.8)
Sclerodactyly without skin thickening proximal to the MCP, N (%)	1005	309 (30.7)
Puffy fingers, N (%)	890	390 (43.8)
mRSS in the last appointment, mean (SD)	752	4.7 (7.1)
Calcinosis, N (%)	983	139 (14.1)
Musculoskeletal disease		
Joints involvement (arthritis or arthralgia), N (%)	1002	400 (39.9)
Flexion contractures, N (%)	953	77 (8.1)
Tendon friction rubs, N (%)	922	23 (2.5)
Myositis, N (%)	984	62 (6.3)
Vascular disease		
Raynaud’s phenomenon, N (%)	1047	978 (93.4)
Digital ulcers, N (%)	1040	365 (35.1)
Pitting scars, N (%)	927	287 (31.0)
Telangiectasia, N (%)	1008	493 (48.9)
Pulmonary arterial hypertension, N (%)	1021	19 (1.9)
Capillaroscopy changes, N (%)	697	547 (78.5)
Early scleroderma pattern, N (%)	540	131 (24.3)
Active scleroderma pattern, N (%)	540	147 (27.2)
Late scleroderma pattern, N (%)	540	82 (15.2)
Lung disease		
Interstitial lung disease, N (%)	870	289 (33.2)
Restrictive syndrome, N (%)	696	31 (4.5)
Lung fibrosis on chest radiograph, N (%)	491	133 (27.1)
Fibrosis on chest CT scan, N (%)	694	109 (15.7)
Ground glass opacities on chest CT scan, N (%)	694	75 (10.8)
Honeycombing on chest CT scan, N (%)	693	33 (4.8)
Gastrointestinal disease		
Oesophageal involvement, N (%)	971	354 (36.5)
Gastric involvement, N (%)	951	116 (12.2)
Intestinal involvement, N (%)	946	51 (5.4)
Heart involvement, N (%)	966	73 (7.6)
Renal involvement		
Scleroderma renal crisis, N (%)	961	18 (1.9)
Autoantibodies		
Anti-nuclear antibodies, N (%)	1031	963 (93.4)
Anti-centromere antibodies, N (%)	1043	569 (54.6)
Anti-topoisomerase I antibodies, N (%)	1036	226 (21.8)
Anti-Pm/Scl antibodies, N (%)	665	31 (4.7)
Anti-U1-RNP antibodies, N (%)	693	28 (4.0)
Anti-RNA polymerase III antibodies, N (%)	806	24 (3.0)
Anti-U3-RNP antibodies, N (%)	643	16 (2.5)
Anti-Th/To antibodies, N (%)	606	13 (2.1)
Anti-Ku antibodies, N (%)	633	12 (1.9)
Anti-U11/U12-RNP antibodies, N (%)	613	3 (0.5)

Abbreviations: ACR—American College of Rheumatology, CT—computed tomography, EULAR—European Alliance of Associations for Rheumatology, MCP—metacarpophalangeal joints, mRSS—modified Rodnan skin score, N—number of patients, NPAD—number of patients with available information, RNA—ribonucleic acid, SD—standard deviation, SSc—systemic sclerosis. Data previously presented in the abstract: Dourado, E.; Freitas, R.; Martins, P.; Saraiva, L.; Santiago, T.; Guimarães, F.; Costa, E.; Esperança Almeida, D.; Dinis, S.; Pinto, A.; et al. Prevalence and clinical associations of different autoantibodies in the Reuma.pt systemic sclerosis cohort: is it all really set in stone? *Ann. Rheum. Dis.* **2022**, *81*, 1475–1476. https://doi.org/10.1136/annrheumdis-2022-eular.3389 [21].

**Table 2:** Summary of selected associations between autoantibody positivity and demographic and clinical factors.

	ACA	ATA	ARA	Th/To	U3RNP	PmScl	Ku	U1RNP	U11/12
**Female sex**	N = 1043, *p* < 0.001 *	N = 1036, *p* = 0.001 **	N = 806, *p* = 0.115	N = 606, *p* = 0.023 **	N = 643, *p* = 0.461	N = 665, *p* = 0.010 **	N = 633, *p* = 0.067	N = 693, *p* = 0.266	N = 613, *p* = 1.000
**Age 1st symptom**	N = 808, *p* < 0.001 *	N = 802, *p* = 0.171	N = 664, *p* = 0.580	N = 509, *p* = 0.566	N = 537, *p* = 0.945	N = 560, *p* = 0.379	N = 527, *p* = 0.813	N = 583, *p* = 0.001 **	N = 520, *p* = 0.304
**Age diagnosis**	N = 911, *p* < 0.001 *	N = 904, *p* = 0.036 **	N = 703, *p* = 0.399	N = 533, *p* = 0.439	N = 563, *p* = 0.487	N = 586, *p* = 0.317	N = 554, *p* = 0.962	N = 613, *p* < 0.001 **	N = 544, *p* = 0.660
**Age last appointment ^†^**	N = 1043, *p* < 0.001 *	N = 1036, *p* = 0.441	N = 806, *p* = 0.038 **	N = 606, *p* = 0.843	N = 643, *p* = 0.489	N = 665, *p* = 0.063	N = 633, *p* = 0.577	N = 693, *p* < 0.001 **	N = 613, *p* = 0.971
**lcSSc**	N = 1043, *p* < 0.001 *	N = 1036, *p* < 0.001 **	N = 806, *p* = 0.323	N = 606, *p* = 0.619	N = 643, *p* = 0.073	N = 665, *p* = 0.568	N = 633, *p* = 0.325	N = 693, *p* < 0.001 **	N = 613, *p* = 1.000
**dcSSc**	N = 1043, *p* < 0.001 **	N = 1036, *p* < 0.001 *	N = 806, *p* = 0.101	N = 606, *p* = 0.139	N = 643, *p* = 0.001 *	N = 665, *p* = 0.259	N = 633, *p* = 0.138	N = 693, *p* = 1.000	N = 613, *p* = 1.000
**ssSSc**	N = 1043, *p* = 0.229	N = 1036, *p* = 0.882	N = 806, *p* = 0.550	N = 606, *p* = 0.065	N = 643, *p* = 1.000	N = 665, *p* = 1.000	N = 633, *p* = 1.000	N = 693, *p* = 0.618	N = 613, *p* = 1.000
**VEDOSS**	N = 1043, *p* = 0.012 *	N = 1036, *p* = 0.008 **	N = 806, *p* = 0.155	N = 606, *p* = 0.471	N = 643, *p* = 0.331	N = 665, *p* = 0.861	N = 633, *p* = 0.234	N = 693, *p* = 0.008 **	N = 613, *p* = 0.431
**2013 CC**	N = 1010, *p* = 0.007 **	N = 1003, *p* < 0.001 *	N = 776, *p* = 0.171	N = 577, *p* = 0.078	N = 614, *p* = 0.083	N = 636, *p* = 0.694	N = 603, *p* = 0.736	N = 664, *p* = 0.053	N = 584, *p* = 1.000
**Diagnosis delay**	N = 800, *p* = 0.031 *	N = 793, *p* = 0.142	N = 656, *p* = 0.107	N = 503, *p* = 0.608	N = 530, *p* = 0.241	N = 553, *p* = 0.460	N = 521, *p* = 0.475	N = 576, *p* = 0.032 **	N = 514, *p* = 0.121
**Disease duration**	N = 808, *p* = 0.066	N = 802, *p* = 0.805	N = 664, *p* = 0.002 **	N = 509, *p* = 0.181	N = 537, *p* = 0.268	N = 560, *p* = 0.494	N = 527, *p* = 0.640	N = 583, *p* = 0.317	N = 520, *p* = 0.083
**White European ancestry**	N = 626, *p* = 0.003 *	N = 620, *p* = 0.115	N = 517, *p* = 0.631	N = 408, *p* = 0.472	N = 440, *p* = 0.557	N = 441, *p* = 0.658	N = 425, *p* = 1.000	N = 449, *p* = 0.029 **	N = 414, *p* = 1.000
**PM**	N = 1042, *p* < 0.001 **	N = 1035, *p* = 0.194	N = 805, *p* = 1.000	N = 605, *p* = 0.025 *	N = 643, *p* = 1.000	N = 664, *p* < 0.001 *	N = 632, *p* = 0.201	N = 693, *p* = 0.442	N = 613, *p* = 1.000
**DM**	N = 1042, *p* = 0.004 **	N = 1035, *p* = 0.080	N = 805, *p* = 0.192	N = 605, *p* = 1.000	N = 642, *p* = 1.000	N = 664, *p* = 0.243	N = 632, *p* = 1.000	N = 692, *p* = 1.000	N = 612, *p* = 1.000
**SLE**	N = 1043, *p* = 0.386	N = 1036, *p* = 0.779	N = 806, *p* = 0.262	N = 606, *p* = 1.000	N = 643, *p* = 1.000	N = 665, *p* = 1.000	N = 633, *p* = 0.014 *	N = 693, *p* = 0.058	N = 613, *p* = 1.000
**SD**	N = 1040, *p* = 0.677	N = 1033, *p* = 0.282	N = 804, *p* = 0.348	N = 604, *p* = 1.000	N = 641, *p* = 1.000	N = 663, *p* = 1.000	N = 631, *p* = 1.000	N = 691, *p* = 0.443	N = 611, *p* = 1.000
**RA**	N = 1043, *p* = 0.522	N = 1036, *p* = 0.382	N = 806, *p* = 1.000	N = 606, *p* = 1.000	N = 643, *p* = 1.000	N = 665, *p* = 1.000	N = 633, *p* = 1.000	N = 693, *p* = 1.000	N = 613, *p* = 1.000
**MCTD**	N = 1043, *p* < 0.001 **	N = 1036, *p* = 0.015 **	N = 806, *p* = 0.158	N = 606, *p* = 1.000	N = 643, *p* = 0.352	N = 665, *p* = 0.601	N = 633, *p* = 0.047 *	N = 693, *p* < 0.001 *	N = 613, *p* = 1.000
**Skin thickening**	N = 1024, *p* < 0.001 **	N = 1018, *p* < 0.001 *	N = 794, *p* = 0.409	N = 596, *p* = 0.236	N = 633, *p* = 0.413	N = 655, *p* = 0.546	N = 623, *p* = 0.358	N = 682, *p* = 0.462	N = 604, *p* = 0.228
**Puffy fingers**	N = 878, *p* < 0.001 **	N = 875, *p* < 0.001 *	N = 670, *p* = 0.132	N = 484, *p* = 0.514	N = 520, *p* = 0.086	N = 539, *p* = 0.606	N = 506, *p* = 1.000	N = 567, *p* = 0.906	N = 494, *p* = 0.502
**mRSS (last appointment)**	N = 736, *p* < 0.001 **	N = 735, *p* < 0.001 *	N = 643, *p* = 0.014 *	N = 507, *p* = 0.479	N = 530, *p* = 0.394	N = 542, *p* = 0.961	N = 525, *p* = 0.687	N = 569, *p* = 0.093	N = 508, *p* = 0.465
**Raynaud’s phenomenon**	N = 1024, *p* = 0.949	N = 1017, *p* = 0.782	N = 792, *p* = 1.000	N = 592, *p* = 0.192	N = 630, *p* = 1.000	N = 651, *p* = 0.055	N = 619, *p* = 0.555	N = 680, *p* = 0.251	N = 602, *p* = 1.000
**Digital ulcers**	N = 1015, *p* = 0.001 **	N = 1010, *p* < 0.001 *	N = 785, *p* = 0.573	N = 586, *p* = 1.000	N = 624, *p* = 0.098	N = 645, *p* = 0.514	N = 613, *p* = 0.763	N = 674, *p* = 0.510	N = 595, *p* = 0.558
**Pitting scars**	N = 912, *p* = 0.008 **	N = 907, *p* < 0.001 *	N = 689, *p* = 0.429	N = 496, *p* = 1.000	N = 534, *p* = 0.130	N = 555, *p* = 0.890	N = 522, *p* = 0.720	N = 582, *p* = 0.508	N = 506, *p* = 1.000
**Telangiectasia**	N = 989, *p* = 0.069	N = 983, *p* = 0.542	N = 767, *p* = 0.961	N = 576, *p* = 0.232	N = 613, *p* = 0.109	N = 633, *p* = 0.014 **	N = 602, *p* = 1.000	N = 660, *p* = 0.364	N = 587, *p* = 0.563
**Joint involvement**	N = 984, *p* = 0.335	N = 978, *p* = 0.577	N = 763, *p* = 0.620	N = 577, *p* = 0.207	N = 614, *p* = 0.668	N = 633, *p* = 0.019 *	N = 602, *p* = 0.555	N = 661, *p* = 0.013 *	N = 585, *p* = 0.289
**Flexion contractures**	N = 936, *p* < 0.001 **	N = 934, *p* < 0.001 *	N = 730, *p* = 1.000	N = 556, *p* = 0.613	N = 588, *p* = 0.100	N = 603, *p* = 1.000	N = 571, *p* = 0.581	N = 633, *p* = 1.000	N = 564, *p* = 1.000
**TFR**	N = 908, *p* = 0.053	N = 906, *p* = 0.004 *	N = 709, *p* = 0.424	N = 535, *p* = 1.000	N = 568, *p* = 1.000	N = 584, *p* = 1.000	N = 553, *p* = 0.232	N = 613, *p* = 0.496	N = 545, *p* = 1.000
**Calcinosis**	N = 965, *p* = 0.649	N = 963, *p* = 0.267	N = 755, *p* = 0.311	N = 572, *p* = 1.000	N = 607, *p* = 0.050 *	N = 626, *p* = 0.023 *	N = 594, *p* = 0.615	N = 653, *p* = 0.333	N = 581, *p* = 1.000
**Myositis**	N = 966, *p* < 0.001 **	N = 963, *p* = 0.316	N = 755, *p* = 1.000	N = 574, *p* = 0.033 *	N = 610, *p* = 0.211	N = 631, *p* = 0.001 *	N = 597, *p* = 0.020 *	N = 657, *p* < 0.001 *	N = 584, *p* = 1.000
**ILD**	N = 853, *p* < 0.001 **	N = 852, *p* < 0.001 *	N = 649, *p* = 0.565	N = 472, *p* = 0.706	N = 508, *p* = 1.000	N = 525, *p* = 0.037 *	N = 491, *p* = 0.706	N = 553, *p* = 0.432	N = 482, *p* = 0.514
**PAH**	N = 992, *p* = 0.191	N = 985, *p* = 1.000	N = 763, *p* = 1.000	N = 567, *p* = 1.000	N = 604, *p* = 1.000	N = 624, *p* = 1.000	N = 592, *p* = 1.000	N = 652, *p* = 1.000	N = 575, *p* = 1.000
**Oesophageal involvement**	N = 950, *p* = 0.037 **	N = 947, *p* = 0.001 *	N = 737, *p* = 0.445	N = 560, *p* = 0.122	N = 595, *p* = 0.092	N = 614, *p* = 0.258	N = 580, *p* = 0.163	N = 642, *p* = 0.410	N = 569, *p* = 0.563
**Heart involvement**	N = 951, *p* = 0.122	N = 948, *p* = 0.016 *	N = 736, *p* = 1.000	N = 556, *p* = 1.000	N = 592, *p* = 1.000	N = 610, *p* = 0.445	N = 576, *p* = 1.000	N = 639, *p* = 0.713	N = 565, *p* = 1.000
**Scleroderma renal crisis**	N = 945, *p* = 0.168	N = 941, *p* = 1.000	N = 735, *p* = 0.007 *	N = 554, *p* = 1.000	N = 591, *p* = 0.018 *	N = 611, *p* = 1.000	N = 579, *p* = 0.081	N = 638, *p* = 0.271	N = 564, *p* = 1.000
**Capillaroscopic changes ^†^**	N = 687, *p* = 0.463	N = 683, *p* = 0.352	N = 556, *p* = 1.000	N = 424, *p* = 1.000	N = 452, *p* = 1.000	N = 463, *p* = 0.954	N = 438, *p* = 1.000	N = 480, *p* = 0.408	N = 429, *p* = 1.000

**Abbreviations:** ACA—anti-centromere antibody, ARA—anti-RNA polymerase III, ATA—anti-topoisomerase I, CC—classification criteria, dcSSc—diffuse cutaneous systemic sclerosis, DM—overlap with dermatomyositis, ILD—interstitial lung disease, Ku—anti-Ku antibodies, lcSSc—limited cutaneous systemic sclerosis, MCTD—mixed connective tissue disease, mRSS—modified Rodnan skin score, N—number of patients with available information for both variables, PAH—pulmonary arterial hypertension, PM—overlap with polymyositis, PmScl—anti-Pm/Scl antibodies, RA—overlap with rheumatoid arthritis, SLE—overlap with systemic lupus erythematosus, SD—overlap with Sjögren’s disease, ssSSc—systemic sclerosis sine scleroderma, TFR—tendon friction rubs, Th/To—anti-Th/To antibodies, U1RNP—anti-U1-RNP antibodies, U11/12—anti-U11/U12-RNP antibodies, U3RNP—anti-U3-RNP antibodies, VEDOSS—very early diagnosis of systemic sclerosis. Bold values indicate associations: * positive association; ** inverse association. ^†^ Variables not considered in the Bonferroni correction.

**Table 3:** Multivariable binary logistic regression analyses of selected clinical manifestations according to autoantibody profile.

	Full Cohort			2013 ACR/EULAR Subgroup		
Clinical Manifestation	ATA vs. ACA Adjusted OR (95% CI); *p* Value	Non-ACA/ATA vs. ACA Adjusted OR (95% CI); *p* Value	ATA vs. Non-ACA/ATA Adjusted OR (95% CI); *p* Value	ATA vs. ACA Adjusted OR (95% CI); *p* Value	Non-ACA/ATA vs. ACA Adjusted OR (95% CI); *p* Value	ATA vs. Non-ACA/ATA Adjusted OR (95% CI); *p* Value
Oesophageal involvement	**1.92 (1.30–2.83); *p* = 0.001**	1.13 (0.76–1.67); *p* = 0.548	**1.70 (1.09–2.64); *p* = 0.018**	**1.58 (1.03–2.41); *p* = 0.036**	1.05 (0.67–1.65); *p* = 0.831	1.50 (0.93–2.43); *p* = 0.100
Calcinosis	1.00 (0.57–1.75); *p* = 0.993	0.90 (0.52–1.55); *p* = 0.694	1.11 (0.59–2.09); *p* = 0.736	0.79 (0.44–1.42); *p* = 0.430	0.75 (0.41–1.38); *p* = 0.361	1.05 (0.54–2.04); *p* = 0.888
PAH	0.91 (0.18–4.75); *p* = 0.914	1.54 (0.40–5.96); *p* = 0.536	0.60 (0.11–3.31); *p* = 0.554	0.79 (0.15–4.14); *p* = 0.776	1.59 (0.40–6.33); *p* = 0.510	0.49 (0.09–2.75); *p* = 0.421
SRC	2.89 (0.61–13.69); *p* = 0.180	3.72 (0.86–16.17); *p* = 0.080	0.78 (0.21–2.83); *p* = 0.703	2.33 (0.48–11.42); *p* = 0.295	3.08 (0.66–14.45); *p* = 0.154	0.76 (0.20–2.91); *p* = 0.686

**Abbreviations:** ACA—anti-centromere antibody, ATA—anti-topoisomerase I antibody, CI—confidence interval, OR—odds ratio, PAH—pulmonary arterial hypertension.

## Data Availability

The data that support the findings of this study are available from the corresponding author upon reasonable request.

## Supplementary Materials

The following supporting information can be downloaded at https://www.mdpi.com/article/10.3390/antib15050085/s1: Table S1: Associations between autoantibody positivity and demographic and clinical factors.
