# Supplementary material for: Prevalence and Clinical Associations of Systemic Sclerosis-Related Autoantibodies: A Nationwide Reuma.pt Cohort Study
Source: Antibodies (Basel). 2026 Sep 15;15(5):85. doi: 10.3390/antib15050085 (PMC13600187; doi:10.3390/antib15050085)
Supplement: Supplementary file 1 [file antibodies-15-00085-s001.zip › antibodies-4474591-supplementary.pdf]

Supplementary files:

Table S1 - Associations between autoantibody positivity and demographic and clinical factors.

	Female sex	Age 1st symptom	Age diagnosis	Age last appointment	lcSSc	dcSSc	ssSSc	VEDOSS	PM	DM	SLE	SD
ACA	N=1043, p<0.001	N=808, p<0.001	N=911, p<0.001	N=1043, p<0.001	N=1043, p<0.001	N=1043, p<0.001	N=1043, p=0.229	N=1043, p=0.012	N=1042, p<0.001	N=1042, p=0.004	N=1043, p=0.386	N=1040, p=0.677
ATA	N=1036, p=0.001	N=802, p=0.171	N=904, p=0.036	N=1036, p=0.441	N=1036, p<0.001	N=1036, p<0.001	N=1036, p=0.882	N=1036, p=0.008	N=1035, p=0.194	N=1035, p=0.080	N=1036, p=0.779	N=1033, p=0.282
ARA	N=806, p=0.115	N=664, p=0.580	N=703, p=0.399	N=806, p=0.038	N=806, p=0.323	N=806, p=0.101	N=806, p=0.550	N=806, p=0.155	N=805, p=1.000	N=805, p=0.192	N=806, p=0.262	N=804, p=0.348
Th/To	N=606, p=0.023	N=509, p=0.566	N=533, p=0.439	N=606, p=0.843	N=606, p=0.619	N=606, p=0.139	N=606, p=0.065	N=606, p=0.471	N=605, p=0.025	N=605, p=1.000	N=606, p=1.000	N=604, p=1.000
U3RNP	N=643, p=0.461	N=537, p=0.945	N=563, p=0.487	N=643, p=0.489	N=643, p=0.073	N=643, p=0.001	N=643, p=1.000	N=643, p=0.331	N=643, p=1.000	N=642, p=1.000	N=643, p=1.000	N=641, p=1.000
PmScl	N=665, p=0.010	N=560, p=0.379	N=586, p=0.317	N=665, p=0.063	N=665, p=0.568	N=665, p=0.259	N=665, p=1.000	N=665, p=0.861	N=664, p<0.001	N=664, p=0.243	N=665, p=1.000	N=663, p=1.000
Ku	N=633, p=0.067	N=527, p=0.813	N=554, p=0.962	N=633, p=0.577	N=633, p=0.325	N=633, p=0.138	N=633, p=1.000	N=633, p=0.234	N=632, p=0.201	N=632, p=1.000	N=633, p=0.014	N=631, p=1.000
U1RNP	N=693, p=0.266	N=583, p=0.001	N=613, p<0.001	N=693, p<0.001	N=693, p<0.001	N=693, p=1.000	N=693, p=0.618	N=693, p=0.008	N=693, p=0.442	N=692, p=1.000	N=693, p=0.058	N=691, p=0.443
U11/12	N=613, p=1.000	N=520, p=0.304	N=544, p=0.660	N=613, p=0.971	N=613, p=1.000	N=613, p=1.000	N=613, p=1.000	N=613, p=0.431	N=613, p=1.000	N=612, p=1.000	N=613, p=1.000	N=611, p=1.000

Abbreviations: ACA – anti-centromere antibodies, ARA - anti–RNA polymerase III, ATA - anti–topoisomerase I, CC – classification criteria, CT – computed tomography, dcSSc – diffuse cutaneous systemic sclerosis, DM – overlap with dermatomyositis, ILD – interstitial lung disease, Ku – anti-Ku antibodies, lcSSc – limited cutaneous systemic sclerosis, LFCR – lung fibrosis on chest radiograph, MCTD – mixed connective tissue disease, mRSS – modified Rodnan skin score, N – number of patients with available information for both variables, PAH – pulmonary arterial hypertension, PM – overlap with polymyositis, PmScl – anti-Pm/Scl antibodies, RA – overlap with rheumatoid arthritis, SLE – overlap with systemic lupus erythematosus, SD – overlap with Sjögren's disease, ssSSc – systemic sclerosis sine scleroderma, STPMCF – skin thickening proximal to the metacarpophalangeal joints, TFR – tendon friction rubs, Th/To – anti-Th/To antibodies, U1RNP – anti-U1-RNP antibodies, U11/12 – anti-U11/U12-RNP antibodies, U3RNP – anti-U3-RNP antibodies, VEDOSS – very early diagnosis of systemic sclerosis. Blue and orange cells represent positive and inverse associations,

respectively. Dark grey represents independent variables that were not considered in the analysis, and were excluded from the Bonferroni correction; SSc subtypes, capillaroscopic patterns, ancestry and skin thickening variables were counted as single non-decomposed variables.

	Joint involvement	Flexion contractures	TFR	Myositis	Raynaud phenomenon	Digital ulcers	Pitting scars	Telangiectasia	PAH	Capillaroscopic changes
ACA	N=984, p=0.335	N=936, p<0.001	N=908, p=0.053	N=966, p<0.001	N=1024, p=0.949	N=1015, p=0.001	N=912, p=0.008	N=989, p=0.069	N=992, p=0.191	N=687, p=0.463
ATA	N=978, p=0.577	N=934, p<0.001	N=906, p=0.004	N=963, p=0.316	N=1017, p=0.782	N=1010, p<0.001	N=907, p<0.001	N=983, p=0.542	N=985, p=1.000	N=683, p=0.352
ARA	N=763, p=0.620	N=730, p=1.000	N=709, p=0.424	N=755, p=1.000	N=792, p=1.000	N=785, p=0.573	N=689, p=0.429	N=767, p=0.961	N=763, p=1.000	N=556, p=1.000
Th/To	N=577, p=0.207	N=556, p=0.613	N=535, p=1.000	N=574, p=0.033	N=592, p=0.192	N=586, p=1.000	N=496, p=1.000	N=576, p=0.232	N=567, p=1.000	N=424, p=1.000
U3RNP	N=614, p=0.668	N=588, p=0.100	N=568, p=1.000	N=610, p=0.211	N=630, p=1.000	N=624, p=0.098	N=534, p=0.130	N=613, p=0.109	N=604, p=1.000	N=452, p=1.000

PmScl	N=633, p=0.019	N=603, p=1.000	N=584, p=1.000	N=631, p=0.001	N=651, p=0.055	N=645, p=0.514	N=555, p=0.890	N=633, p=0.014	N=624, p=1.000	N=463, p=0.954
Ku	N=602, p=0.555	N=571, p=0.581	N=553, p=0.232	N=597, p=0.020	N=619, p=0.555	N=613, p=0.763	N=522, p=0.720	N=602, p=1.000	N=592, p=1.000	N=438, p=1.000
U1RNP	N=661, p=0.013	N=633, p=1.000	N=613, p=0.496	N=657, p<0.001	N=680, p=0.251	N=674, p=0.510	N=582, p=0.508	N=660, p=0.364	N=652, p=1.000	N=480, p=0.408
U11/12	N=585, p=0.289	N=564, p=1.000	N=545, p=1.000	N=584, p=1.000	N=602, p=1.000	N=595, p=0.558	N=506, p=1.000	N=587, p=0.563	N=575, p=1.000	N=429, p=1.000

	RA	MCTD	2013 CC	1980 CC	Skin thickening	STPMCF in any location	STPMCF in hand	Sclerodactyly without STPMCF	Puffy fingers	mRSS (last appointment)	Calcinosis
ACA	N=1043, p=0.522	N=1043, p<0.001	N=1010, p=0.007	N=882, p<0.001	N=1024, p<0.001	N=887, p<0.001	N=988, p<0.001	N=985, p<0.001	N=878, p<0.001	N=736, p<0.001	N=965, p=0.649
ATA	N=1036, p=0.382	N=1036, p=0.015	N=1003, p<0.001	N=876, p<0.001	N=1018, p<0.001	N=882, p<0.001	N=983, p<0.001	N=980, p=0.001	N=875, p<0.001	N=735, p<0.001	N=963, p=0.267
ARA	N=806, p=1.000	N=806, p=0.158	N=776, p=0.171	N=665, p=0.488	N=794, p=0.409	N=685, p=0.183	N=762, p=0.083	N=760, p=0.379	N=670, p=0.132	N=643, p=0.014	N=755, p=0.311
Th/To	N=606, p=1.000	N=606, p=1.000	N=577, p=0.078	N=477, p=0.276	N=596, p=0.236	N=503, p=0.339	N=570, p=0.129	N=569, p=0.717	N=484, p=0.514	N=507, p=0.479	N=572, p=1.000
U3RNP	N=643, p=1.000	N=643, p=0.352	N=614, p=0.083	N=514, p=0.178	N=633, p=0.413	N=539, p=0.508	N=607, p=0.468	N=606, p=0.551	N=520, p=0.086	N=530, p=0.394	N=607, p=0.050
PmScl	N=665, p=1.000	N=665, p=0.601	N=636, p=0.694	N=535, p=0.399	N=655, p=0.546	N=557, p=0.620	N=629, p=0.323	N=628, p=0.651	N=539, p=0.606	N=542, p=0.961	N=626, p=0.023
Ku	N=633, p=1.000	N=633, p=0.047	N=603, p=0.736	N=503, p=1.000	N=623, p=0.358	N=527, p=0.334	N=596, p=0.336	N=595, p=1.000	N=506, p=1.000	N=525, p=0.687	N=594, p=0.615
U1RNP	N=693, p=1.000	N=693, p<0.001	N=664, p=0.053	N=564, p=0.030	N=682, p=0.462	N=583, p=0.470	N=655, p=0.485	N=654, p=0.561	N=567, p=0.906	N=569, p=0.093	N=653, p=0.333
U11/12	N=613, p=1.000	N=613, p=1.000	N=584, p=1.000	N=485, p=0.164	N=604, p=0.228	N=512, p=0.249	N=579, p=0.272	N=578, p=0.557	N=494, p=0.502	N=508, p=0.465	N=581, p=1.000

	Early pattern	Active pattern	Late pattern	Normal capillaroscopy	ILD	Restrictive syndrome	LFCR	Fibrosis on CT	Ground glass on CT	Honeycombing on CT
ACA	N=532, p=0.658	N=532, p=0.394	N=532, p=0.580	N=532, p=0.430	N=853, p<0.001	N=684, p<0.001	N=484, p<0.001	N=682, p<0.001	N=682, p<0.001	N=681, p<0.001
ATA	N=530, p=0.130	N=530, p=0.029	N=530, p=0.818	N=530, p=0.395	N=852, p<0.001	N=683, p<0.001	N=482, p<0.001	N=681, p<0.001	N=681, p<0.001	N=680, p<0.001
ARA	N=442, p=1.000	N=442, p=0.533	N=442, p=1.000	N=442, p=1.000	N=649, p=0.565	N=518, p=0.514	N=335, p=0.189	N=516, p=0.711	N=516, p=1.000	N=515, p=1.000
Th/To	N=371, p=0.660	N=371, p=1.000	N=371, p=1.000	N=371, p=0.682	N=472, p=0.706	N=393, p=0.180	N=185, p=0.675	N=393, p=1.000	N=393, p=1.000	N=392, p=1.000
U3RNP	N=375, p=1.000	N=375, p=1.000	N=375, p=0.231	N=375, p=0.489	N=508, p=1.000	N=418, p=0.092	N=218, p=0.340	N=417, p=1.000	N=417, p=0.609	N=416, p=1.000
PmScl	N=382, p=0.372	N=382, p=0.119	N=382, p=0.401	N=382, p=0.308	N=525, p=0.037	N=427, p=1.000	N=235, p=0.142	N=426, p=1.000	N=426, p=1.000	N=425, p=1.000
Ku	N=373, p=0.461	N=373, p=0.404	N=373, p=1.000	N=373, p=0.523	N=491, p=0.706	N=402, p=1.000	N=202, p=1.000	N=402, p=1.000	N=402, p=1.000	N=401, p=1.000
U1RNP	N=394, p=0.493	N=394, p=0.470	N=394, p=0.620	N=394, p=0.350	N=553, p=0.432	N=448, p=0.238	N=256, p=0.166	N=447, p=0.328	N=447, p=0.405	N=446, p=0.605
U11/12	N=364, p=1.000	N=364, p=0.400	N=364, p=1.000	N=364, p=1.000	N=482, p=0.514	N=401, p=0.052	N=198, p=1.000	N=400, p=1.000	N=400, p=1.000	N=400, p=1.000

	Oesophageal involvement	Gastric involvement	Intestinal involvement	Heart involvement	Scleroderma renal crisis	Diagnosis delay	Disease duration	White European ancestry	African ancestry
ACA	N=950, p=0.037	N=936, p=0.282	N=930, p=0.065	N=951, p=0.122	N=945, p=0.168	N=800, p=0.031	N=808, p=0.066	N=626, p=0.003	N=626, p=0.014
ATA	N=947, p=0.001	N=935, p=0.064	N=929, p=0.117	N=948, p=0.016	N=941, p=1.000	N=793, p=0.142	N=802, p=0.805	N=620, p=0.115	N=620, p=0.148
ARA	N=737, p=0.445	N=729, p=1.000	N=722, p=1.000	N=736, p=1.000	N=735, p=0.007	N=656, p=0.107	N=664, p=0.002	N=517, p=0.631	N=517, p=1.000
Th/To	N=560, p=0.122	N=559, p=1.000	N=554, p=0.468	N=556, p=1.000	N=554, p=1.000	N=503, p=0.608	N=509, p=0.181	N=408, p=0.472	N=408, p=0.333
U3RNP	N=595, p=0.092	N=591, p=1.000	N=586, p=0.124	N=592, p=1.000	N=591, p=0.018	N=530, p=0.241	N=537, p=0.268	N=440, p=0.557	N=440, p=0.404
PmScl	N=614, p=0.258	N=607, p=1.000	N=601, p=0.634	N=610, p=0.445	N=611, p=1.000	N=553, p=0.460	N=560, p=0.494	N=441, p=0.658	N=441, p=0.614
Ku	N=580, p=0.163	N=574, p=0.302	N=568, p=0.334	N=576, p=1.000	N=579, p=0.081	N=521, p=0.475	N=527, p=0.640	N=425, p=1.000	N=441, p=1.000
U1RNP	N=642, p=0.410	N=636, p=0.236	N=630, p=0.308	N=639, p=0.713	N=638, p=0.271	N=576, p=0.032	N=583, p=0.317	N=449, p=0.029	N=449, p=0.007
U11/12	N=569, p=0.563	N=566, p=1.000	N=561, p=1.000	N=565, p=1.000	N=564, p=1.000	N=514, p=0.121	N=520, p=0.083	N=414, p=1.000	N=414, p=1.000
